# Thyroid Autoimmunity and Lichen

**DOI:** 10.3389/fendo.2017.00146

**Published:** 2017-06-27

**Authors:** Fabrizio Guarneri, Roberta Giuffrida, Flavia Di Bari, Serafinella Patrizia Cannavò, Salvatore Benvenga

**Affiliations:** ^1^Department of Clinical and Experimental Medicine – Dermatology, University of Messina, Messina, Italy; ^2^Department of Clinical and Experimental Medicine – Endocrinology, University of Messina, Messina, Italy; ^3^Master Program on Childhood, Adolescent and Women’s Endocrine Health, University of Messina, Messina, Italy; ^4^Interdepartmental Program of Molecular & Clinical Endocrinology and Women’s Endocrine Health, University Hospital Policlinico “G. Martino”, Messina, Italy

**Keywords:** autoimmune thyroid disease, oral lichen planus, mucous lichen planus, cutaneous lichen planus, lichen sclerosus, human leukocyte antigen, infections, molecular mimicry

## Abstract

Lichen planus (LP) and lichen sclerosus (LS) are cutaneous-mucous diseases with uncertain epidemiology. Current data, which are likely to be underestimated, suggest a prevalence in the general population of 0.1–4% for cutaneous LP, 1.27–2.0% for oral LP, and 0.1–3.3% for LS. While etiology of lichen is still unknown, clinical and histological evidence show an (auto)immune pathogenesis. Association of lichen with autoimmune thyroid disease (AITD) has been investigated in few studies. This association appears better defined in the case of LS, while is more controversial for LP. In both situations, the frequency of the association is higher in females. We review the available literature on the correlation between the different types of lichen and AITD, and the literature on the genetic risk factors which are shared by both conditions. Such data suggest that a common pathogenic mechanism could be the cause for co-occurrence of lichen and AITD, at least in some patients. Additionally, analyzing literature data and in continuity with our previous work on other autoimmune diseases, we suggest that molecular mimicry could trigger both diseases, and thus explain their co-occurrence.

## Introduction

Lichen planus (LP) is a cutaneous-mucous disease characterized by small papules and pruritus. Histologically, LP is characterized by hyperkeratosis, hypergranulosis, acanthosis with formation of colloid bodies (Civatte or Sabouraud bodies), vacuolar degeneration of the basal epidermal cell layer, “saw-tooth” appearance of the rete pegs, enlarged and deformed dermal papillae with infiltration of lymphocytes and histiocytes in a “band-like” pattern in contact with, and sometimes invading, the basal epidermal cell layer (1). Because of clinical similarity, over the years, several dermatological conditions were defined as “lichen” to eventually form the so-called lichenoid dermatoses, but with different specifications. For instance, lichen striatus is actually classified among spongiotic dermatoses, lichen amyloidosus among amyloidoses, and lichen aureus among mucinoses (1). A detailed discussion about lichenoid dermatoses is beyond the scope of this review. Only LP (cutaneous, mucosal and oral) and lichen sclerosus (LS)—also known as LS and atrophicus—for which autoimmunity has been postulated as a relevant part of the pathogenic mechanism, were reported to be associated with autoimmune thyroid disease (AITD).

The epidemiology of LP and LS is not exactly defined. Current data, probably underestimated, suggest a prevalence in the general population of 0.1–4% for cutaneous LP (2), 1.27–2.0% for oral LP (3), and 0.1–3.3% for LS (4). Few authors investigated the association between these conditions and thyroid diseases and, even fewer, the possible common etiopathogenic mechanism(s).

We, herein, review the available literature on this topic. Moreover, in continuity with our previous studies on molecular mimicry as a trigger of autoimmune diseases, we suggest molecular mimicry as a potential pathogenic mechanism.

## Materials and Methods

We searched the PubMed database (https://ncbi.nlm.nih.gov/PubMed) using the search string “thyroid AND autoimm* AND lichen.” The retrieved articles were revised, and only those discussing the association between lichen and AITD were selected. The reference lists of these articles were examined to find other relevant articles, which were also revised and included in this review if appropriate.

## Results

As of February 24, 2017, a PubMed search for “AITD” or “lichen” retrieved 14,864 and 15,311 articles, respectively. Conversely, a search for “thyroid AND autoimm* AND lichen,” as described in Section “Material and Methods,” yielded 40 articles of which 17 were selected as they were relevant for our review. Of these 17 articles, 15 analyzed the occurrence of AITD in patients with lichen (Table 1) and 2 analyzed the occurrence of lichen in patients with AITD (Table 2).

**Table 1:** Studies reporting clinical and/or laboratory data about subjects affected by lichen who were found positive for AITD.

Reference, *Country*	Type of study and population	Main findings
Goolamali et al. (5), *England*	Cross-sectional study on 26 patients with **LS** (25 females, 1 males) and 443 controls without autoimmune diseases	10/25 (40%) of female patients were positive for antithyroid cytoplasm antibodies (*p* < 0.001 vs controls); 8 of these 10 patients had subclinical thyroiditis

Tremaine et al. (6), *Canada*	Case report	Report on a 65-year-old woman simultaneously affected by **LS**, AITD, morphea, and insulin-dependent diabetes mellitus

Kreuter et al. (12), *Germany*	Retrospective prevalence study on 532 patients affected by **LS** (396 females, 136 males)	AITD was associated with **LS** in 65 cases (12.2% of patients), prevalently among females [*n* = 60 (15.2%), *p* = 0.0002 vs males]

Cooper et al. (13), *England*	Case–control study on 190 women with adult-onset vulvar **LS**, 126 women with adult-onset erosive vulvar **LP**, 922 age-matched control women	AITD was observed in 16.3% of patients with **LS** and 15% of patients with erosive **LP** vs 7.9% of controls (*p* < 0.001 for both comparisons)

Kazandi et al. (14), *Turkey*	Retrospective prevalence study on 82 women with vulvar **LS**	15 cases of thyroid disease were observed (18.2% of patients)

Birenbaum and Young (15), *USA*	Retrospective prevalence study on 211 women with vulvar **LS**	63 cases of AITD were observed (29.9% of patients). Prevalence of AITD was higher among patients aged less than 55 years [25/74 (33.8%)] than those aged 55 years or more [38/137 (27.7%)]

Kantere et al. (16), *Sweden*	Cross-sectional study on 100 patients with **LS**	No patients with thyroid disease, five with mild abnormality of thyroid function: low levels of T4 and raised levels of TSH in two cases, normal T4 and raised TSH in two other cases, normal T4 and decreased TSH in one

Hagedorn et al. (18), *Germany*	Cross-sectional study on 102 patients (60 females, 42 males) with **LS**	AITD was found in 39% of females and 12.5% of males

Ebrahimi et al. (20), *Sweden*	Cross-sectional study on 120 patients (89 females, 31 males) with mucosal **LP** and 87 age- and sex-matched healthy controls	AITD was found in 11/120 patients (9.2%)

Chang et al. (21), *Taiwan*	Cross-sectional study on 500 patients with desquamative gingivitis, 287 with erosive oral **LP** without desquamative gingivitis, and 100 healthy controls	455 patients with desquamative gingivitis were affected by erosive oral **LP**; 46.4% of them were positive for anti-Tg and 45.1% for antithyroid microsomal antibodies. The corresponding percentages were 27.5 and 30.3%, among patients with erosive **LP** but not gingivitis. For both groups of patients, differences in comparison with healthy controls were significant (*p* < 0.001). Overall, 210 patients were positive for at least one thyroid autoantibody, and TSH levels were normal in 84.3%, decreased in 6.7% and increased in 9% of them

Chang et al. (22), *Taiwan*	Cross-sectional study on 320 patients with oral **LP** (292 erosive, 28 non-erosive)	Anti-Tg and antithyroid microsomal antibodies were found in 21.3 and 24.4% of patients. TSH levels were normal in 85.8% of the 190 patients, positive for one or both thyroid autoantibodies, below normal in 4.2% and above normal in 10%

Carrozzo et al. (23), *Italy*	Cross-sectional study on 50 patients with oral **LP**	Antithyroid antibodies were found in 10% of patients

Lavaee and Majd (24), *Iran*	Retrospective prevalence study on 523 patients with oral **LP** (387 females, 136 males) and 523 age- and sex-matched healthy controls	Hypothyroid subjects were 35 among patients (6.7%) and 21 among controls (4%); this difference was not statistically significant

Azurdia et al. (31), *England*	Cross-sectional study on 58 males affected by **LS** and 602 healthy controls	*HLA-DR11, -DR12*, and *-DQ7* were significantly (*p* ≤ 0.05) more frequent in patients, but no cases of AITD were found among them. One patient had a mild increase of serum T4 with normal TSH levels, another one had slightly lower than normal serum T4, with normal TSH. A third patient had positive antithyroid antibodies, but normal thyroid function

Aslanian et al. (32), *Brazil*	Cross-sectional study on three families (30 subjects in total) with familial **LS**	8 cases of **LS** were found among the 30 subjects visited; seven of these patients were positive for anti-TPO antibodies, but only four had clinical AITD. The HLA-B*15-DRB1*04-DRB4* haplotype was associated with the co-occurrence of **LS** and thyroid autoimmunity

*The type of lichen studied in each paper is written in boldface*.

*AITD, autoimmune thyroid disease; LP, lichen planus; LS, lichen sclerosus; T4, thyroxine; Tg, thyroglobulin; TPO, thyroid peroxidase; TSH, thyroid stimulating hormone; HLA, human leukocyte antigen*.

**Table 2:** Studies reporting clinical and/or laboratory data about subjects affected by AITD who were found positive for lichen.

Reference, *country*	Type of study and population	Main findings
Soy et al. (19), *Turkey*	Cross-sectional study on 65 patients (56 females, 9 males) with AITD	Oral **LP** was found in two patients (3.1%)

Bra˘nis¸teanu et al. (25), *Romania*	Retrospective prevalence study on 38 patients (36 Females, 2 males) with thyroid diseases (63% autoimmune thyroiditis, 26.3% polynodular goiter, 10.7% hypothyroidism)	**LP** was found in 18% of patients and was the second most frequent dermatological disorder after alopecia areata (22%)

*The type of lichen studied in each paper is written in boldface*.

*AITD, autoimmune thyroid disease, LP, lichen planus*.

The first paper which suggested that LS “may be related to or caused by an autoimmune process” and highlighted the frequent association with thyroid autoimmunity was published in 1974 (5). In 26 patients (25 females and one male) with LS and 443 control subjects without autoimmune diseases, the authors evaluated the presence of antibodies to thyroglobulin (Tg), thyroid cytoplasm, gastric parietal cells, and type I intrinsic factor. Almost half of the female patients were positive for antithyroid cytoplasm antibodies [10/25 (40%), *p* < 0.001 vs controls] or anti-gastric parietal cells antibodies [11/25 (44%), *p* < 0.001 vs controls]; other tests were negative or not different from controls. Eight patients with antithyroid cytoplasm antibodies had subclinical thyroiditis (5).

The topic was brought again to attention many years later, in a case report of a 65-year-old woman with coexisting LS, AITD, morphea, and insulin-dependent diabetes mellitus (6). Next, a comment (7) to a review article (8) provocatively defined LS “a cutaneous manifestation of thyroid disease” (7). While this statement appears excessive in the light of modern knowledge, it is suggestive of the increase, started in the early 1990s, of awareness and interest concerning the possible connection between lichen and AITD (9–11). Larger studies, needed to define the epidemiological relevance of the association, have been performed only relatively recently.

### LS and AITD

The largest, yet retrospective, study available on LS and AITD is on 532 patients with LS, predominantly adults and females (*n* = 500 and 396, respectively), who were visited in a German University hospital during 12 years (12). All patients were examined for cutaneous and extracutaneous autoimmune diseases. Tests included complete blood cell count, routine blood chemistry testing, complement components 3 and 4, C-reactive protein, and a panel of serological analyses for autoimmunity. This panel included antinuclear antibodies, extractable nuclear antibodies, rheumatoid factor, anti-cyclic citrullinated peptide antibodies, circulating immune complexes, and antibodies to thyroid peroxidase (TPO) and Tg. One or more autoimmune diseases were found in 82 patients (15.4%), with a significantly (*p* < 0.0001) different frequency among women (*n* = 75, 18.9%) and men (*n* = 7, 5.1%). AITD [Hashimoto’s thyroiditis (HT) or Graves’ disease (GD)] was the condition most often associated with LS, with a total of 65 cases (12.2%), again prevalently among females [*n* = 60 (15.2%), *p* = 0.0002 vs males]. Overall, cases of all other autoimmune diseases accounted for 3.3% of the studied sample.

Similar results were found by others. A case–control study in women was performed (13) on a smaller population (190 with adult-onset vulvar LS, 126 with adult-onset erosive vulvar LP, 922 age-matched controls). AITD was observed in 16.3% of patients with LS and 15% of patients with erosive LP compared to 7.9% in controls (*p* < 0.001 for both comparisons) (13). Kazandi et al. (14) retrospectively analyzed 82 women with vulvar LS and found 15 cases of thyroid disease (18.2%). A greater prevalence (29.9%) was reported in a retrospective evaluation of 211 patients visited in a 10-year period for vulvar LS (15). The percentage was higher among patients aged less than 55 years (33.8%) than among those aged 55 years or more (27.7%).

A different picture can be observed in studies on male LS. After the aforementioned article by Kreuter et al. (12), the largest and probably most complete study was published by Kantere et al. (16). The authors randomly chose 100 patients from 771 diagnosed with LS between 1997 and 2007 and re-evaluated their clinical condition. Such re-evaluation included clinical examination and laboratory tests, namely thyroid stimulating hormone (TSH), thyroxine (T4), antinuclear antibodies, and autoantibodies to extracellular matrix protein-1 (ECM-1), ECM-1 being the likely autoantigen of LS (17). Only five patients had mild abnormal thyroid function: low levels of T4 and raised levels of TSH (which is consistent with overt primary hypothyroidism) in two cases, normal T4 and raised TSH (which is consistent with subclinical primary hypothyroidism) in two other cases, normal T4 and decreased TSH in one (16). However, one paper (18) shows a different trend. Indeed, in a population of 60 women and 42 men, Hagedorn et al. (18) found that LS was associated with AITD in 39% of female and 12.5% of male patients.

### LP (Oral, Mucous, Cutaneous) and AITD

The association between thyroid autoimmunity and LP, in its different subtypes, is more controversial, and the scarcity of papers does not allow clear conclusions. Soy et al. (19) evaluated the frequency of rheumatic and autoimmune diseases in 65 patients (56 women and 9 men) with AITD. Oral LP was one of the less represented diseases, as it was found in only two patients (3.1%). Ebrahimi et al. (20) evaluated 120 patients with mucosal LP and 87 age- and sex-matched healthy controls for the presence of other diseases, with particular attention to autoimmune and thyroid diseases. They found a significantly high frequency of autoimmune diseases in general (28%) and AITD in particular (9.2%) among patients. These results, together with the observation that lichen was multifocal in 72% of women and 64% of men, led the authors to conclude that “LP with mucosal involvement should be considered and taken care of as a systemic disease” and to point out “the need for a multidisciplinary clinic to get optimal care and treatment” (20). Another paper concerning mucosal lichen, namely adult-onset erosive vulvar LP, is by Cooper et al. (13) (see above, Section “LS and AITD”). Further, interesting elements come from a recent paper (21). This paper (21) aimed to define the number of patients with desquamative gingivitis (a disease often associated with erosive oral LP) who were positive for anti-gastric parietal cells, anti-Tg, and antithyroid microsomal antibodies. They analyzed 500 patients with desquamative gingivitis, 287 with erosive oral LP but without desquamative gingivitis, and 100 healthy controls. Upon careful reevaluation, erosive oral LP was found in 455 patients of the first group: 46.4% of them were positive for anti-Tg and 45.1% for antithyroid microsomal antibodies. The percentages were 27.5 and 30.3%, respectively, among patients of the second group. Differences from controls were significant (*p* < 0.001) for both groups of patients. Overall, 210 patients were positive for at least one thyroid-related autoantibody, and TSH levels were normal in 84.3%, low in 6.7%, and raised in 9% of them (21). In a previous study (22), the same group had found a prevalence of 21.3 and 24.4% for anti-Tg and antithyroid microsomal antibodies, respectively, among 320 patients with oral LP (erosive in 292 cases, non-erosive in 28). In the same study, TSH levels were normal in 85.8% of the 190 patients positive for one or both thyroid-related autoantibodies, low in 4.2% and raised in 10% (22). Other authors had reported antithyroid antibodies in 10% of 50 patients with oral LP (23).

Lavaee and Majd (24) retrospectively evaluated the frequency of hypothyroidism in 523 patients with oral LP (387 females, 136 males) and in an equal number of age- and sex-matched healthy controls. They found statistically similar proportions (6.7 and 4%, respectively).

Finally, Brănişteanu et al. published data about the association between AITD and cutaneous LP (25). The study population included 38 patients (36 females, 2 males) with thyroid diseases (63% autoimmune thyroiditis, 26.3% multinodular goiter, 10.7% hypothyroidism), who accessed the Dermatovenereology Unit of a University hospital over 2 years. LP was the second most frequent dermatological disorder observed (18%) after alopecia areata (22%).

### Genetic Risk Factors: Possible Role of Human Leukocyte Antigen (HLA)

The studies mentioned in the previous sections suggest that all subtypes of lichen, AITD, and also their association, are more frequent in females.

Given the autoimmune pathogenesis of both conditions, several authors analyzed the possibility of a link with specific alleles of the *HLA* genes. As well known, *HLA* genes generate the major histocompatibility complex (MHC) molecules, responsible for presentation of (auto)antigenic peptides to the immune system and activation of the consequent specific (auto)immune reaction.

Studies on the *HLA* haplotype of patients with lichen are few, not very recent, and often performed on small cohorts. Porter et al. (26) reported that cutaneous LP has been associated to *HLA-A3, -A5, -A28, -B16*, and *-Bw35*, mucosal LP to *HLA-A3* and *-A28*, oral LP to *HLA-B16, -DR1*, and *-DRw9*, erosive oral LP to *HLA-DR2, -DR3, -DR9, -B27*, and *-Bw57*, mixed oral LP to *HLA-B51* [for references, see Ref. (26)]. The studies reviewed had populations ranging from 10 to 82 patients, and had been published between 1976 and 1994. For LS, the most recent review (27) suggests a strong linkage to *HLA-DQ7*. In a subsequent case–control study (28), an increased frequency of HLA-DRB1*12/DQB1*03 was found in 187 patients with vulvar LS.

A comparison with AITD-associated *HLA* alleles (29) shows some elements in common: *HLA-B16* confers increased risk for HT in Asians, *HLA-DR3* is linked to GD (in Caucasians) and HT, *HLA-DR9* is a risk factor for GD in Japanese and Chinese subjects and HT in Chinese patients only [for references, see Ref. (29)]. Among patients with stress-related GD, *HLA-A28* is significantly more frequent (at least 3-X) in those with exacerbations of hyperthyroidism compared with those with no exacerbations during treatment with antithyroid drugs, while *HLA-DR3* is almost 3-times more frequent in the whole group of patients with stress-related GD compared with healthy controls (30).

The above data could suggest a common genetic background of susceptibility for lichen and thyroid autoimmunity. However, we found only two studies that evaluated the *HLA* haplotypes of patients for which the association between lichen and AITD was explicitly investigated (31, 32). Azurdia et al. (31) analyzed 58 males with LS and 602 healthy controls, and showed a significantly (*p* ≤ 0.05) higher frequency of *HLA-DR11, -DR12*, and *-DQ7* in patients. In detail, the frequencies of *HLA-DR11, -DR12*, and *-DQ7* were 22, 9, and 45% among patients and 13, 3, and 31% among controls, respectively. Abnormal thyroid function was observed in two cases: one patient had a mild increase of serum T4 with normal TSH levels, while another had slightly subnormal serum T4, and normal TSH. Positive antithyroid antibodies, but normal thyroid function, were found in a third patient (31). In the second paper, Aslanian et al. (32) examined three families, of 20, 8, and 2 members, respectively, with familial LS. Eight subjects with LS were found among the 30 visited, 7 of whom were positive for anti-TPO antibodies, but only 4 had a thyroid disease. The HLA-B*15-DRB1*04-DRB4* haplotype was associated with the co-occurrence of LS and thyroid autoimmunity (32). HLA-DRB1*04 was almost threefold more frequent in patients with stress-related GD compared with healthy controls (30).

### Environmental Triggering Factors: Association with Infections and the Molecular Mimicry Hypothesis

Like most autoimmune diseases, the environmental triggers of LS and AITD are unknown, and also unknown is whether an etiopathogenic link between the two conditions exists.

We previously reported a woman who developed both LS and HT after infection by *Borrelia burgdorferi* (33). In that occasion, the chronological sequence and correlation between the pathological events led us to hypothesize that molecular mimicry between bacterial antigen(s) and human autoantigens could have been the pathogenic mechanism by which borreliosis had triggered both autoimmune diseases (33). According to the molecular mimicry hypothesis, structural similarity between microbial antigens and human autoantigens can turn a defensive immune reaction into an autoimmune reaction in genetically predisposed subjects (mainly because of specific *HLA* alleles). This model has been postulated, and in many cases demonstrated, as a possible explanation for the onset of autoimmunity (34–41).

Several studies on the possible role of molecular mimicry in the pathogenesis of autoimmune and allergic diseases were performed also by our group, with extensive use of bioinformatics tools (42–53). In detail, we searched for amino acid sequence homology between human protein autoantigens involved in specific autoimmune diseases and proteins from microbes that are clinically linked to such diseases. In many cases, we also searched the homologous segments of human and microbial proteins for binding motifs of MHC molecules derived from specific *HLA* alleles.

Following the hypothesis formulated in our case report (33), we aimed to identify the molecules most probably involved in triggering autoimmunity after *Borrelia* infection (42, 45). We found that human TSH-R has four segments homologous to proteins from *Borrelia* and five homologous to proteins from *Yersinia*, another bacterial species associated with AITD. In a subsequent study (45), we extended our work to include the other known thyroid autoantigens (TPO, Tg, sodium iodide symporter) and to search human and microbial proteins for the occurrence of peptide-binding motifs of *HLA-DR* molecules. Eleven additional homologies were found with proteins from *Borrelia* (2 with Tg, 3 with TPO, 6 with sodium iodide symporter) and 15 with proteins from *Yersinia* (2 with Tg, 2 with TPO, 11 with sodium iodide symporter). The number of binding motifs related to the different *HLA-DR* alleles agreed well with literature data, which suggest that AITD is associated with *HLA-DR3, -DR4, -DR5, -DR8*, and *-DR9*.

Concerning the association between *Borrelia* and lichen, in 1985, Asbrink wrote that “a *Borrelia* infection may result in lichen sclerosus et atrophicus-like reactions” (54), a claim that was subsequently supported by others (55–59). Although the debate remains open, a pathogenic link between borreliosis and lichen seems to exist in some cases (60). Our preliminary data (61) show that ECM-1, which is the autoantigen of LS (17), is homologous to BBG23 and methyl-accepting chemotaxis protein (mcp-3) of *B. burgdorferi*. All four thyroid autoantigens, ECM-1, and their corresponding homologous *Borrelia* proteins contain 4–32 copies of the binding motif related to *HLA-DQ7*, this allele conferring genetic susceptibility to both AITD (62) and LS (31).

Molecular mimicry appears as an interesting field of investigation, and might explain, at least in part, associations found in epidemiological studies and/or single case reports. In our experience, it gave a plausible explanation for the association between AITD and *Yersinia* infection (42, 45, 50), anti-tumor vaccination with NY-ESO-1 (51), or rickettsiosis (52).

The main other infectious agents that the literature has linked to AITD are Epstein–Barr virus (63), hepatitis C virus (64), parvovirus B19 (64), human herpesvirus-6 (65), and *Helicobacter pylori* (66). For lichen, association was reported with Epstein–Barr virus—also known as human herpesvirus 4 (67), hepatitis C virus (67, 68), human papillomavirus (67, 69), and human herpesvirus-7 (70), while correlation with *H. pylori* is controversial (71, 72).

## Conclusion

The existence and nature of a connection between AITD and lichen are still unresolved issues. Echoing Braun-Falco et al. (1), the etiology of lichen is currently “a mistery.” Molecular mimicry is a likely mechanism, especially considering the advantage of providing an explanation for the occurrence of the association in patients with given *HLA* genotypes. However, molecular mimicry alone may not explain entirely the complex pathogenesis of the association, and other possibilities should be evaluated. Better awareness and attention to the association of lichen and AITD, and increased interdisciplinary collaboration, is desirable to define epidemiological magnitude and detailed clinical characteristics of the association, taking into account variables, such as ethnicity, socioeconomic issues, and environmental issues. Hopefully, better basic and clinical research will generate more effective care to patients with coexisting lichen and AITD.

## Author Contributions

SB and FG: substantial contributions to the conception or design of the work, drafting of the work, final approval of the version to be published, and agreement to be accountable for all aspects of the work in ensuring that questions related to the accuracy or integrity of any part of the work are appropriately investigated and resolved. SPC, FDB, and RG: acquisition/analysis/interpretation of data for the work, critical revision for important intellectual content, final approval of the version to be published, and agreement to be accountable for all aspects of the work in ensuring that questions related to the accuracy or integrity of any part of the work are appropriately investigated and resolved.

## Conflict of Interest Statement

The research was conducted in the absence of any commercial or financial relationships that could be construed as a potential conflict of interest.

## Abbreviations

AITD, autoimmune thyroid disease; GD, Graves’ disease; HT, Hashimoto’s thyroiditis; LP, lichen planus; LS, lichen sclerosus; T4, thyroxine; Tg, thyroglobulin; TPO, thyroid peroxidase; TSH, thyroid stimulating hormone.
